# What are the lived career experiences of senior operating department practitioners? An interpretative phenomenological analysis

**DOI:** 10.1177/17504589251319967

**Published:** 2025-02-24

**Authors:** Lee Rollason

**Affiliations:** College of Health and Care Professions, Birmingham City University, Birmingham, UK

**Keywords:** Operating department practitioner, ODP, Lived experiences, Career experiences, Workplace culture, Visibility, Phenomenology

## Abstract

Operating department practitioners are registered health care practitioners who provide specialist care to patients across the perioperative environment in the disciplines of anaesthetics, surgery and post-anaesthetic care. There is a significant knowledge gap that surrounds the profession in terms of their career experiences. Using a methodology of interpretative phenomenological analysis, the lived career experiences of six senior operating department practitioners were investigated. Seven themes were identified across the experiences of participants addressing a wide range of topics including issues such as professional development, relationships with other health care professionals, sexual safety, student experiences and visibility. Recommendations based on this study include further investigation of operating department practitioner experiences across a wider demographic, an immediate review of how job adverts are constructed to be more inclusive and a call for increased professional visibility of operating department practitioners. Long-term recommendations include a resolution around the use of Patient Group Directives, workplace culture reviews and a review of theatre-attire appropriateness in relation to sexual safety in the perioperative environment.

## Introduction

Operating department practitioners (ODPs) are registered health care practitioners regulated by the Health and Care Professions Council (HCPC) and a member of the Allied Health Professionals (AHPs). ODPs specialise in the provision of health care to patients within the perioperative environment working in three core areas: anaesthetics, surgery and post-anaesthetic care. Although ODPs work mainly in surgical settings, they are increasingly found in areas such as intensive care units and advanced clinical practice (Intensive Care Society 2024, National Health Service (NHS) England 2024a).

Although there is a substantive history behind ODPs and their previous incarnations as theatre technicians and then operating department assistants, there is a knowledge gap around ODPs as a modern professional group (College of Operating Department Practitioners (CODP) 2021).

Exploring the lived career experiences of ODPs is important as it can provide an enhanced understanding of important issues that relate to ODPs. Understanding factors like the challenges that ODPs face in their careers and the factors that influence their day-to-day practice will allow for an improved perspective on issues such as staff retention and thereby the capacity to improve service provision within perioperative environment.

## Literature review

### Operating department practitioners and nursing

Before regulation in 2004, accounts of ODPs in practice were often provided by nursing colleagues rather than ODPs themselves. These accounts have negative connotations and highlight tension and conflict between the ODPs and nurses of the time (Timmons & Tanner 2004). Williams (2008) provides one such account where they detail workforce division, territorial disputes and the perceptions of power and status between the two groups.

Despite reports of conflict, nurses and ODPs have found common ground through their shared goals and objectives of patient care within the perioperative environment leading to the widely used professional title ‘Perioperative Practitioner’ for any registered health care practitioner working in the perioperative department (Eviota 2021). This in itself may be indicative of a process of homogenisation of professional cultures within the perioperative environment.

The emerging harmony between staff groups may be attributed to mutual appreciation of specialised training of ODPs and that accrued by nurses with experience in surgery. The current undergraduate nursing curriculum does not require a nurse to be proficient in perioperative practice (Nursing and Midwifery Council 2018). However, newly qualified nurses are able to practice in theatre upon qualifying, despite not having any experience. This seems to lead to feelings of inexperience and not being prepared, especially when compared with the ODP, who is regarded as a specialist in the field even upon first qualifying (Stone 2019). Indeed, there is evidence that nurses may feel unprepared to work in the operating theatres with their undergraduate studies alone, as some undertake secondary training in Operating Department Practice (Pinchbeck 2011).

### The emerging student voice

While qualitative accounts from ODPs are relatively few, students or newly qualified practitioners appear keener to describe their experiences of training (Rodger & Mahoney 2017, Westmoreland 2020, Young 2012). However, when accounts turn to career experience – or expectations of careers – wording is often vague and lacks detail in respect of career progression or opportunities (Critchlow 2010). A possible explanation for the lack of detailed expectations within those undergoing training in Operating Department Practice may be due to a lack of exposure to the career which starts at earlier ages. Hewkin (2008), for example, investigated the attitudes of young people (12–18 years of age) towards ODPs. The outcome suggested that among the sample group, there was little understanding of what an ODP was or what their duties might include, this was attributed in part to the visibility of health care roles within modern media.

### The future of operating department practice

The lack of literature surrounding the future careers of the ODP makes it difficult to draw conclusions as to what that future might hold, although there seems to be a tendency towards cautious optimism (Cadman 2017). There is the suggestion that there is a place for advanced clinical practice among perioperative practitioners and ODPs are being included as part of investigations into advanced practitioner training (Quick 2010, Wallis et al 2022).

It was the aim of this study to build upon existing evidence and to give a voice to ODPs in respect of their own lived career experiences, which seems to be lacking. In turn, that evidence can be used to garner insight and move towards understanding and improvement for the profession.

## Methods

### Methodology

This study utilised a qualitative paradigm (Given 2008). The study was undertaken from an ontological positionality of relativism and contextualism (Eastwood et al 2018). Contextualism is a middle-ground between positivism and constructionism that promotes an ideology of accepting universal truths but not necessarily separating them from subject experience (Byrne & Ragin 2009). Contextualism was chosen on the basis that there are constructs within the experience of being an ODP that are the same for all ODPs and not necessarily open to interpretation, for example, sharing a common professional history, being registered with the HCPC and having to abide by standards of proficiency, but there is also space in which the individual ODP constructs meaning and significance of their own career on a personal and subjective level. The chosen methodology was Interpretative Phenomenological Analysis (IPA) (Smith et al 2022). IPA was chosen because it was necessary for the participants to speak about their experiences in a way that was meaningful for them, determining what experiences were important and to what extent, and to make sense of those experiences for themselves. This allowed the researcher access to data that was meaningful to the participant. In addition, IPA acknowledges the research as an interpretative mechanism in the analytical process and facilitates the role of the researcher as someone who is able to identify themes between participant accounts that may not be readily identifiable by any single participant themselves.

### Ethical considerations

Prior to commencing this research, ethical approval was sought from Birmingham City University’s ethics committee as part of a post-graduate Master Research programme. Ethical approval was granted on 31 May 2023 (Ref: Rollason/011850/sub2/*R*(A)/2023/May/HELS FAEC). Detailed ethical considerations were given to all stages of the research process ensuring the safeguarding of all participants throughout and these are detailed as follows:

### Population and sampling

Participants were recruited through the use of social media (X, formerly known as Twitter). Participants were required to have a minimum of 1 year experience post-qualification, this stipulation was made to ensure that participants had sufficient lived career experiences to call upon when discussing their careers as ODPs. Newly qualified ODPs, those in post for less than 1 year, are likely to have limited experiences as an ODP which would in turn limit the data that could be captured. There was no requirement to have an ongoing registration with the HCPC to capture retired ODPs or those with lapsed registrations. Participants were excluded if currently involved in fitness to practice (FTP) proceedings so as not to compromise the interviewer and/or researcher in respect of what might be disclosed during the interview. This is not to say that those undergoing FTP proceedings do not have valid experiences to contribute, but those experiences were beyond the consideration of this study.

A total of 19 people volunteered to take part. After receiving an information pack 12 people confirmed they wanted to proceed. Due to the timeline constraints of this study, of those 12 people, six participants were randomly selected. The chosen participants underwent the consent process before the interviews were arranged. Random selection was necessary to avoid the introduction of selection bias.

### Data collection and analysis

Participants were invited to a semi-structured interview which took place via an online platform. All interviews lasted approximately 1 h. Interviews were then transcribed and made ready for analysis. Manual data analysis was carried out in accordance with the IPA techniques described by Braun and Clarke (2013). This process involved the coding of each transcript across three different areas of interest: description, linguistics and concepts. The codes that were generated were collected from all transcripts and were explored for common thoughts, ideas and views. Resultingly, similar codes were grouped, and emerging themes were identified and named accordingly.

The researcher himself is an ODP and though every effort was made to ensure that the data spoke for itself through staying closely aligned with the selected methodology, the potential for bias cannot be entirely eliminated.

## Results

### Theme 1: I believe this is central to being an operating department practitioner

Participants identified a number of personal and professional aspects which informed their core identity as ODPs. For some this related to patient care while others focused on more technical abilities or outcome-based values:

P1A: ‘I think ODPs are very, we’re quite scientific, we’re quite meticulous with our work . . .’
P2B: ‘it comes down to that quality of care really [pauses] I could do better than that’.
P4D: ‘if I could contribute to a service, to patient care, well, you know, if I think I can do that, I could do that, and I’ll go for it’.

### Theme 2: Growing (or not) as an operating department practitioner

This theme emerged around the participant’s reflections that related directly to the career of an ODP. This theme highlighted challenges that the participants felt that they had experienced in their careers including being limited in terms of progression and feeling left out. Often this happens because people outside of the operating department do not know who or what ODPs are and are therefore not inclusive:

P1A: ‘So, in terms of going up the ladder in theatres, you reach that glass ceiling’.
P3 C: ‘It’s just a language that’s, that’s used erm that unfortunately sometimes the language that’s used erm just eradicates us from er being part of what’s being discussed’.
P5E: ‘without sounding rude, why? This is me, these are my qualifications, this is my background, why am I not suitable? And she went “hmm, that’s a good point, you are!”’

### Theme 3: How I feel about working with other professions

The third theme formed around participants’ experiences with other health care professionals. This revealed feelings of inequality and frustration for the ODP when compared with other health care professions, particularly nursing:

P1A: ‘. . . travesty because we are the experts in that area and nurses will come in and they’ll learn how to scrub, but they don’t have the depth of understanding that we have because our three-year degree is specifically for theatres’.
P3 C: ‘We’re just trying to be on an, an equal footing! We’re just asking for equality in the workplace, you know’.
P4D: ‘all I’ve ever wanted was a [sic] equality of opportunity for my profession’.

### Theme 4: My feelings about the culture I work in

Theme four reports on participants’ experiences of ODP working environments including workplace culture, which was inconsistent both within the same organisation and often between different organisations. Workplace safety, particularly psychological and sexual safety were also highlighted within this theme:

P1A: ‘so I think for me, definitely the workplace, workplace culture is probably one of the most negative’.
P1A: ‘And I think probably because I was a female and because these were internationally educated doctors as well and they had made a couple of comments about me being a woman in charge’.
P2B: ‘And most of us as well seem to have a really good sense of humour, so you know, and you, you know, you can, and it’s part of, it’s just part of the camaraderie and the banter’.
P6 F: ‘I actually start to think about, you know, the, the V cut tops that we had, and the comments, maybe, that were, you know, and, and actually how, what I would listen to and put up with now to what I did then would be quite different, I think’.

### Theme 5: When I was a student ODP

Theme five focused on participants’ experiences as students. This theme reflected a difficult university experience and both the powerful effect, yet inconsistent quality of mentors:

P1A: ‘I remember producing a poster and getting 98% and thinking well, where’s the feedback? What did I lose the 2% for and being told I didn’t like the colour [pauses] it would have been much more productive to have more time in practise because [stammers] I felt I er missed out a lot on laparoscopic work’.
P1A: ‘And when I told them I wasn’t taking a job there, I was called into the office and told that I had stabbed them in the back and let them down’.
P2B: ‘and I was being supervised – already from the coffee room – by somebody’.
P6 F: ‘I’ve had a couple of very good clinical educators who have took me under their wing really and, and seen my potential’.

### Theme 6: Raising our flag

The sixth theme arose around the visibility of the profession, starting with the commonality that none of the participants knew what an ODP was prior to a meeting of happenstance. This theme developed into discussing the lack of knowledge around ODPs by other clinical professions and many of the frustrations around visibility were levelled at the CODP:

P1A: ‘I think the challenge for ODPs is that it is completely overshadowed by nursing and erm there is a real lack of awareness of the profession’.
P3 C: ‘if I’m being honest, CODP is a very poor representative’.
P5E: ‘but I wouldn’t be able to tell you who any of the other members were in terms of their sort of job descriptions or anything like that, I just went “I want to work in an operating theatre, do you have to be a nurse?”’

### Theme 7: PGDs and me

The seventh theme reflects the participants’ concerns around ODPs accessing PGDs. Participants expressed feelings of concern in being held back in this regard and that despite the promises of action that have been made, there had been no movement on this issue for what seemed like years:

P2B: ‘Definitely the thing that holds it back is all of the restrictions, the political stuff er the PGDs. All of that’.
P5E: ‘but I feel like I was having these conversations as a student and when I [hesitates] 20 something years down the line and it’s still, it’s still an issue’.

## Discussion

All ODPs who took part in this study discussed patient interaction with positivity and satisfaction; however, they also identified intrinsic elements such as being technically minded, scientific and outcome-based which continue to reflect some of the older characteristics of the workforce as described by Williams (2008). This may be indicative of a continuing process whereby ODPs are still transitioning into holistic health care practitioners, or perhaps the profession is more attractive to people with strengths in technical or scientific skills.

Regardless of how participants interpreted what makes a good ODP, their experiences suggested that they wanted to remove themselves from the notion that ODPs are an assistive workforce. The reasons offered for the perceptions around being an assistant stem from limited career progression and the fact that independent clinical practice is limited due to proximity working with surgeons and anaesthetists. Instead, ODPs want to be known as independent practitioners within their scope of practice, and as perioperative experts.

Education was a factor commonly discussed by participants in this study, suggesting that pre- or post-education was lacking. For example, academic skills were prioritised over clinical placement for some participants, while others suggested that they did not feel prepared to be a mentor until they had undertaken a self-funded, post-graduate qualification. Participants recognised the need for academia in their work as ODPs but implied that this has not been pitched at the appropriate level. Participants also indicated that their formative experiences in education were both inconsistent and, in some situations, intimidating. Understanding negative experiences is important due to the high level of attrition seen among undergraduate students studying health care courses (Hamshire et al 2019, Rowland & Trueman 2024).

While course content and student experience are important elements to consider in the future of ODP education, so is the way in which academic and placement demands are balanced with student’s free time. More students are having to work to make up the difference between the cost of living and their student loans which will undoubtedly have an impact on university and placement attendance in the future (National Union of Students 2024). Nonetheless, the role of education is important in relation to upskilling the ODP workforce and this may help them transition from feelings of academic inferiority and unpreparedness towards becoming more rounded practitioners who are able to provide dynamic leadership and engagement across the perioperative setting (Barradell 2022, Diggele et al 2020). This may be aided by the fact that ODP education, from 2024, will move away from either a diploma of higher education or a degree, to a degree only model of delivery (Health and Care Professions Council (HCPC) 2021).

Participants in the study had strong feelings about a sense of apathy and a lack of engagement within the profession, something which they had either direct or indirect experience of. Apathy was represented by one participant as being a threat towards the progress of ODPs and suggested that it may even cause a professional regression if not addressed. Increasing engagement among ODPs is likely to result in better career satisfaction, although it is acknowledged that some ODPs may already be in a self-perpetuating cycle around low engagement and low levels of job satisfaction (Gkliati & Saiti 2022).

While not the only predictor of job satisfaction, enjoyable workplace cultures seem to share a positive correlation with job satisfaction and it is important to learn the lessons of both good and bad workplace culture as this is linked to staff retention (Long et al 2023, Mohamed et al 2022, Sheng-Shiung et al 2024). The participants highlighted a mixed experience of workplace culture, some suggesting it was very good, while others discussed the renowned toxic nature of working within the NHS. All female participants reported issues around safety when working in the perioperative environment. Sometimes this took the form of dialogue in which males attempted to make females feel inferior, while others discussed aspects of theatre attire and the ill-fitting and/or revealing nature. Concerns around sexual safety in the perioperative environment have previously drawn the attention of the Royal College of Surgeons (Fleming & Fisher 2021). While calls for improvements in sexual safety have been well received and supported by both the government and NHS, the NHS framework for dealing with sexual safety does not go as far as to address the specific issue of theatre attire (Newlands et al 2024, NHS England 2024b).

Even when ODPs are engaged with their careers, they find it difficult to progress due to a lack of professional visibility outside of the operating theatre and this is compounded by feelings of professional inequality. The lived experience of inequality was proposed by one participant in this study to be a result of organisations such as the National Association of Theatre Nurses attempting to suppress ODPs in the past. It is noted, however, that professional organisations have become increasingly inclusive. The Association for Perioperative Practice, for example, started welcoming ODP membership as soon as ‘ODP’ became a protected professional title. Other participants talked about being managed by nurses who did not understand the role of the ODP or hiring managers who do not include HCPC registered staff on job applications. Not being able to apply for jobs that they are technically capable of doing and which lie within their scope of practice, simply due to the wording of a job advert, was a cause for low morale and anger within participants.

It is important to acknowledge, however, that the dispute between nurses and ODPs exists at a higher abstract level and does not appear to interfere with day-to-day operations. All participants acknowledged that nurses are teammates, allies and mentors in the perioperative environment with some accrediting their successes to the advice of nursing colleagues.

Nurses were not the only professional body to occupy a negative space in the account of participants, some frustration was also directed at the CODP who were not seen to be providing value, support or appropriate representation:

P2B: ‘I think as a body the College of ODPs isn’t, I know there’s, there’s plans afoot isn’t there because we don’t get anything out of it’.
P3 C: ‘if I’m being honest, CODP is a very poor representative’.
P5E: ‘there isn’t quite so much at the moment [stammers] of the College support. I think that [stammers] I know they’re planning on getting better and I hope it does’.

PGDs and independent prescribing remain important issues for the ODPs who took part in this study. For them, it was seen as one of the critical issues that was causing stagnation within the profession:

P2B: ‘Definitely the thing that holds it back is all of the restrictions, the political stuff er the PGDs all of that’.
P5E: ‘but I feel like I was having these conversations [about PGDs] as a student and when I [hesitates] 20 something years down the line and it’s still, it’s still an issue’.

While it is acknowledged that some work has been done in this regard participants expressed a fear of falling behind other colleagues in terms of development and career opportunities due to a lack of resolution on this front (HCPC 2020, NHS England 2020).

ODPs take pride in working closely with patients, however, how they measure success or satisfaction in their careers beyond that seems to be changeable. ODP education is inconsistent and did not necessarily meet the needs of participants when they were studying, some of whom felt underprepared for qualified life. Workplace culture is impactful for ODPs, both positively and negatively with female staff in particular encountering negative experiences. ODPs do have access to career pathways but this group of participants felt overlooked, misunderstood or simply blocked from applying for positions. This seems to be compounded by what participants felt is a lack of professional visibility, conflict with the nursing profession and limited clinical options due to not having PGD access. It is suggested that this may be leading to a sense of apathy within the profession.

## Limitations

Following random sampling, participants shared common demographics leading to very high homogeneity within the group. All participants were white, 30 years old or older, heterosexual and occupied a Band 6 position, or higher, within the NHS. Such similarity between group members suggests significant limitations in terms of generalising or transferring any findings to the wider population of ODPs due to low levels of representation. It should be noted, however, that generalisation is not always the primary aim of qualitative research, and that data saturation can be achieved in relatively few interviews (Drisko 2024, Hennink & Kaiser 2022). It is acknowledged that the themes identified within this study may only be representative of its narrow demographic group and that future studies should look to include more diverse sampling within the study population.

## Conclusion

The ODP is an important health care practitioner, supporting patients during surgery and beyond. This study has identified themes that address some of the knowledge gaps around the profession. ODPs value high-quality care but they are also methodical, scientific practitioners. They are keen to represent themselves as perioperative experts and actively seek to distance themselves from the notion that they are an assistive workforce. There is still some tension between ODPs and nurses – as a profession rather than on an individual basis – due to the ways in which their specialities often transect. While ODPs do have access to career pathways, often access to such careers is limited by a number of factors. The participants in this research suggested that increasing visibility of the profession would improve career prospects which in turn might address feelings of apathy within the profession. Participants also considered that there is work to do by employers and the CODP to improve professional visibility, improve working conditions and find a resolution to issues that have dogged the profession for several years.

## Recommendations

Themes should be explored with larger and more diverse populations, adding to or changing themes as appropriate. Historical accounts from ODPs working prior to regulation are important in addressing the knowledge gap and producing a more rounded view of ODP-nurse relations. There should be further exploration of healthy interprofessional teams, role boundaries and the potential impact of initiatives to improve teamwork and cultures of safety in ODPs’ career lived experiences. Employers should explore the inclusivity of job adverts for registered staff. Long-term strategies need to be developed for the resolution of issues surrounding visibility and PGDs. A review of theatre attire, its design and appropriateness should be considered as part of the NHS’s strategy to deal with sexual safety in the workplace.
